# Utilizing gas flux from automated head chamber systems to estimate dietary energy values for beef cattle fed a finishing diet

**DOI:** 10.1093/jas/skae167

**Published:** 2024-06-12

**Authors:** Jarret A Proctor, Jason K Smith, Nathan S Long, Stacey A Gunter, Vinícius N Gouvêa, Matthew R Beck

**Affiliations:** Department of Animal Science, Texas A&M University, College Station, TX, USA; Department of Animal Science, Texas A&M University, College Station, TX, USA; Texas A&M AgriLife Extension, Department of Animal Science, Texas A&M University, Amarillo, TX, USA; Department of Animal Science, Texas A&M University, College Station, TX, USA; Oklahoma and Central Plains Agricultural Research Center, United States Department of Agriculture, Agricultural Research Service, El Reno, OK, USA; Department of Animal Science, Texas A&M University, College Station, TX, USA; Texas A&M AgriLife Research, Department of Animal Science, Texas A&M University, Amarillo, TX, USA; Conservation and Production Research Laboratory, United States Department of Agriculture, Agricultural Research Service, Bushland, TX, USA

**Keywords:** energetics, finishing beef cattle, heat production, respiratory quotient

## Abstract

Dietary net energy for maintenance (**NE**_**m**_) and gain (**NE**_**g**_) can be estimated using calculations based on live performance or adjusted-final body weight, which is calculated based on carcass characteristics. These values are commonly referred to as performance-adjusted (**pa**) NE_m_ (**paNE**_**m**_) and NE_g_ (**paNE**_**g**_). The NE_m_ and NE_g_ of a diet can also be estimated by adding recovered energy (**RE**) with heat production (**HP**) derived from an automated head chamber system (**AHCS**), which we will term gas-adjusted (**ga**) NE_m_ (**gaNE**_**m**_) and NE_g_ (**gaNE**_**g**_). Furthermore, HP from the Brouwer equation requires an estimate of urinary nitrogen (**UN**) excretion, which can be calculated based on N intake, blood urea N, UN concentration, and urine creatinine, or it could be zeroed. Alternatively, HP can be calculated using an alternative equation based on the respiratory quotient. Demonstrating agreement between pa and ga derived dietary energy values provides an opportunity to validate using the AHCS for energetic experiments and this comparison has not been conducted previously. Accordingly, the objective of this experiment was to assess the agreement between live and carcass paNE_m_ and paNE_g_ with gaNE_m_ and gaNE_g_, where HP was calculated using 4 different approaches. Estimates of HP were not different (*P* = 0.99) between the 4 approaches employed, indicating that all options investigated are appropriate. Live paNE_m_ and paNE_g_ had a higher agreement (Lin’s concordance correlation coefficient [**CCC**] = 0.91) with gaNE_m_ and gaNE_g_ than carcass values (CCC ≤ 0.84). These results suggest that researchers can implement the AHCS to provide good estimates of dietary energy values in finishing beef cattle that are unrestrained.

## Teaser

Automated head chamber systems provide researchers with the ability to estimate gas flux, heat production, and dietary energy values in finishing beef cattle.

## Introduction

Indirect respiration calorimetry has historically been utilized to evaluate gas flux and provide measurements for energy content of ingredients and diets, or energetic efficiency of beef cattle. However, whole-body respiration calorimetry systems require animal restraint in sealed chambers (Blaxter and Waiman, 1964; Blaxter and Wainman, 1966; Wedegaertner and Johnson, 1983). Efforts have been made to adapt whole-body open-circuit calorimetry principles to ventilated headboxes (Delfino and Mathison, 1991; Place et al., 2011) or facemasks (Carstens et al., 1997), and while these methods do not enclose the whole animal in sealed chambers, they still restrict movement. Measurements of gas flux from these systems, including carbon dioxide (CO_2_) and methane (CH_4_) emissions and oxygen (O_2_) consumption, are used to calculate heat production (**HP**) using the equation of Brouwer (1965), and ultimately arrive at estimates of energy utilization or dietary energy content. Yet, these sampling procedures are laborious, expensive, and reduce dry matter intake (**DMI**) relative to when the animals are not restrained (Hammond et al., 2015; Llonch et al., 2018). Thus, one could argue whether indirect calorimetry systems that isolate and restrain animals provide representative information about what is occurring in production environments. Accordingly, other methods to assess the energy content of ingredients and diets or the efficiency of energy utilization by cattle in their production environment should be explored.

Automated head chamber systems (**AHCS**; GreenFeed, C-Lock, Inc., Rapid City, SD) are growing in popularity in the research community (Gunter and Beck, 2018). These systems provide the opportunity to measure gas flux from unrestrained cattle in their production environments. The AHCS can be equipped with sensors to measure CO_2_, CH_4_, and more recently O_2_ (Gunter et al. 2017), and therefore estimates are available to calculate HP using the Brouwer (1965) equation. Furthermore, an equation reported by Kaufmann et al. (2011) was proposed as an alternative means to calculate HP which uses respiratory quotient (**RQ**) and CO_2_ emissions. The RQ is the ratio of respired CO_2_ to consumed O_2_, which are components of carbohydrate and fat metabolism (Blaxter, 1962; Brouwer, 1965). Kaufmann et al. (2011) stated that their equation for HP was derived by modifying the Brouwer (1965) equation and the omission of the urinary N adjustment and has been used by several researchers to estimate HP from cattle while using the AHCS (Pereira et al., 2015; Caetano et al., 2017; Holder et al., 2022). Regardless, using any method to estimate HP with the addition of recovered energy (**RE**) yields an estimate of metabolizable energy (**ME**) intake, which can further be used to approximate dietary net energy for maintenance (**NE**_**m**_) and gain (**NE**_**g**_).

Estimates of dietary NE_m_ and NE_g_ and subsequent utilization by beef cattle can also be evaluated through observed performance (Owens and Hicks, 2019). Because of the mathematical relationship between metabolizable and net energy, researchers have estimated dietary NE_m_ and NE_g_ using quadratic equations (Owens et al., 1984; Zinn and Shen, 1998; Zinn et al., 2003, 2008; Vasconcelos and Galyean, 2008). Metrics of DMI, average daily gain (**ADG**), body weight (**BW**), and required NE_m_ and NE_g_ are incorporated into quadratic equations to provide a solution for performance-adjusted NE_m_ (**paNE**_**m**_) and NE_g_ (**paNE**_**g**_). Perhaps to provide a more accurate assessment of paNE_m_ and paNE_g_, Owens and Hicks (2019) suggested using an adjusted-final body weight (**AFBW**) based on carcass adiposity as described by Guiroy et al. (2001). One limitation associated with paNE_m_ and paNE_g_ is that calculating required NE_m_ and NE_g_ is a component of the quadratic equations and, as noted by Owens and Hicks (2019), cattle with lower or higher NE_m_ requirement may elicit variation in residual NE_m_ and NE_g_.

Gas flux collected from AHCS units deployed in feedlot pens may allow evaluation of dietary NE_m_ and NE_g_ in conditions that are favorable to their production environments without prolonged confinement or restriction. Calculating required NE_m_ and NE_g_ values is not a necessary component when using gas flux and RE to forward calculate dietary NE_m_ and NE_g_. Moreover, data from AHCS potentially provides researchers with the opportunity to evaluate HP across dietary treatments. Yet, a comparison should be conducted between values generated from quadratic equations using observed animal performance (Zinn et al., 2008) and those derived from gas-adjusted NE_m_ (**gaNE**_**m**_) and NE_g_ (**gaNE**_**g**_) using gas flux data obtained from an AHCS. Thus, the objective of this experiment was to evaluate estimates of NE_m_ and NE_g_ derived from either live performance, carcass data, or gas flux values. An additional objective of this experiment was to evaluate precision, accuracy, and agreement between methods of calculating performance- and gas-estimated dietary energy values. These different methods include using live weight or AFBW to determine paNE_m_ and paNE_g_ and using the Kaufmann et al. (2011) equation or the Brouwer (1965) equation with or without adjustments for estimated urinary nitrogen (**UN**) excretion to calculate HP. The final objective of this experiment was to simulate a scenario where DMI is unknown and to demonstrate agreement between daily energy intake values determined using the AHCS with daily energy intake values derived from performance-adjusted values. It was hypothesized that gas flux data generated from AHCS would have excellent agreement with performance-adjusted dietary energy values.

## Materials and Methods

This experiment was conducted at the joint USDA-ARS and Texas A&M AgriLife Research feedlot in Bushland, TX. All animal procedures outlined herein were preapproved by the West Texas A&M University Institutional Animal Care and Use Committee (protocol number—2022.01.002).

### Animals and diets

Predominately *Bos taurus*, mix-breed steers (*n *= 54; BW at receiving = 484.1 ± 26.03 kg; mean ± standard deviation) were processed on day −54 with a topical dose of cyfluthrin (Cylence; Elanco Animal Health, Greenfield, IN) and an oral dose of albendazole (Valbazen; Zoetis Animal Health, NJ). Initial hip-height at receiving was 122.7 ± 2.43 cm (mean ± standard deviation). Additionally, steers received (Merck Animal Health; Rahway, NJ) Cavalry 9, Once PMH SQ, and Vista 5 SQ vaccines and were implanted with Revalor-XS. On day −54 steers were randomly assigned to one of two pens, each containing feed bunks designed to measure individual feed intake utilizing an electronic identification system (Calan gate; American Calan, Northwood, NH). Steers were trained to Calan gates for 35 d until day −20 during which time they received a starter diet formulated to contain (on a dry matter [**DM**] basis) 41.0% steam-flaked corn, 19.0% wheat hay, 16.0% corn stalks, 10.8% dried corn distiller’s grains, 6.0% molasses, 5.0% vitamin and mineral supplement, 1.5% corn oil, and 0.7% urea. Once trained, steers were stratified by BW and then randomly assigned to one of three finishing diets (*n* = 18 steers/diet) which were formulated as a component of a separate project (Table 1). Cattle were grouped into 2 pens and each treatment was equally represented in each pen (*n* = 9 per treatment per pen). Treatment diets were formulated to be isoenergetic but vary in crude protein (**CP**) content and analyzed nutritive content of the diets are presented in Table 2. Steers were transitioned from days −20 to 0 utilizing a two-ration blend system where proportions of starter and assigned finishing diets were adjusted until steers were fully transitioned to finishing diets. Steers were fed once daily at 0700 ± 0015 hours and orts were collected prior to feeding for determination of DMI. Finishing diets were fed for 80 d prior to shipment to a commercial abattoir (Tyson Fresh Meats; Amarillo, TX). During the final 28 d on feed, cattle received a daily dose (300 mg/hd) of ractopamine HCl (Optaflexx 45; Elcano Animal Health). Carcass data was collected from each steer by the West Texas A&M University Beef Carcass Research Center.

**Table 1.** Ingredient composition of finishing rations fed to steers for determination of paNE_g_ and paNE_g_ or gaNE_m_ and gaNE_g_

Item, % DM^1^	Ration 1	Ration 2	Ration 3
Steam-flaked corn	76.84	78.08	79.24
Corn stalks	10.00	10.00	10.00
Supplement^2^	5.00	5.00	5.00
Molasses	5.00	3.64	2.33
Corn oil	1.46	1.42	1.38
Urea	1.15	1.48	1.82
Dried distiller’s grain with solubles, corn	0.55	0.26	0.00
Potassium chloride	0.00	0.12	0.23

^1^Ingredients are reported on a DM basis and final rations were adjusted to 75% DM with water.

^2^Formulated supplement composition (DM basis): 27.3611% calcium carbonate, 22.6140% ground corn, 20.6645% magnesium sulfate, 17.3068% monocalcium phosphate, 7.0210% added salt, 4.0344% potassium chloride, 0.3354% Rumensin 90 (Elanco Animal Health), 0.2495% manganese sulfate, 0.1433% vitamin E (500 IU/g), 0.1382% zinc oxide, 0.0798% copper sulfate, 0.0426% sodium selenite, 0.0056% vitamin A (1,000,000 IU/g), 0.0015% cobalt carbonate, 0.0012% ethylenediamine dihydroiodide, 0.0011% vitamin D (500,000 IU/g).

**Table 2.** Analyzed and calculated chemical composition of finishing rations and automated head chamber system bait pellets fed to steers for determination of paNE_g_ and paNE_g_ or gaNE_m_ and gaNE_g_

Items^1^^,^^2^	Ration 1	Ration 2	Ration 3	Ration SEM	Alfalfa Pellet	Pellet SEM
DM, % as fed	74.14	74.11	74.19	0.306	92.82	0.221
CP, % DM	10.77	11.55	12.51	0.107	19.50	0.090
ADF, % DM	8.28	8.82	8.34	0.149	31.30	0.214
NDF, % DM	15.70	15.73	15.79	0.206	42.70	0.298
ADICP, % ADF	16.26	15.51	16.25	0.735	12.20	0.381
NDICP, % NDF	9.11	8.80	9.22	0.375	10.25	0.268
EE, % DM	3.96	3.99	3.97	0.154	2.24	0.113
Ash, % DM	5.54	5.69	5.87	0.129	11.05	0.186
Lignin, % DM	1.68	1.25	1.33	0.130	5.85	0.162
TDN, % DM^3^	87.4	88.2	88.3	0.524	59.0	0.310
NE_m_, Mcal/kg DM^4^	2.21	2.23	2.23	0.021	1.28	0.011
NE_g_, Mcal/kg DM^4^	1.52	1.53	1.53	0.018	0.71	0.007

^1^SFC = steam-flaked corn; DDGS = dried corn distillers’ grains plus solubles; DM = dry matter; CP = crude protein; ADF = acid detergent fiber; NDF = neutral detergent fiber; ADICP = acid detergent insoluble crude protein; NDICP = neutral detergent insoluble crude protein; EE = ether extract; TDN = total digestible nutrients; NE_m_ = net energy for maintenance; NE_g_ = net energy for gain.

^2^Ingredients are reported on a DM basis and rations were adjusted to 75% DM with water, respectively.

^3^TDN calculated utilizing Weiss et al. (1992) model.

^4^NE_m_ and NE_g_ calculated from Galyean et al. (2016) and NASEM (2016).

### Sample collections and measurements

Animals were weighed prior to feeding on days 0, 1, 49, 50, 79, and 80, and the average BW from the paired weight measurements was used for determination of ADG and reported herein. On days 1, 49, and 79, blood was collected into 10-mL blood serum tubes via coccygeal venipuncture. Serum tubes were centrifuged (1,250 × *g*) for 30 min at 4 °C and separated serum was aliquoted into 2-mL microcentrifuge tubes and stored at −20 °C until further analysis. Serum urea nitrogen (**SUN**) concentrations were evaluated using a spectrophotometer (490 nm; BioTek Synergy2 Plate Reader, Agilent Technologies, Santa Clara, CA), and an assay kit (Invitrogen; ThermoFisher Scientific, Waltham, MA) designed for SUN determination (Catalog: EIABUN).

Each pen contained an AHCS for evaluation of daily gas flux. Animals were trained to the AHCS during adaptation to Calan gates and dietary treatments. The AHCS functions similarly to head respiration chambers (Place et al., 2011), but instead measures spot samples and reports daily averages (g/d) for CO_2_ and CH_4_ emissions and O_2_ consumption (Gunter and Beck, 2018). Animals were baited to the AHCS utilizing alfalfa pellets (Hi-Pro Feeds; Friona, TX). Units were calibrated weekly and CO_2_ recoveries were measured on days 1, 40, and 80 to validate unit efficacy. Average CO_2_ recoveries were 98.03 ± 1.64% across all recoveries. Twenty individual drops of bait pellets from each AHCS unit were collected and weighed on days 1, 40, and 80 and each drop averaged 31.6 ± 2.28 g, which was used to calculate AHCS pellet DMI. Units were set to dispense bait pellets in 24-s intervals with 8 drops during each visit, a maximum of 4 visits daily, and a minimum of 4 h between visits. Only visits >3 min in duration were utilized as a component of average daily spot sampling and all animals achieved 30 or more visits lasting 3 min or longer during the 80-d feeding period (Arthur et al., 2017; Gunter and Beck, 2018; Beck et al., 2024).

### Diet chemical composition and analyses

Diet samples were collected daily and composited by week. Samples of the AHCS pellets were collected on days 1, 40, and 80. Daily orts samples and diet and pellet subsamples were dried in triplicate in a forced-air drying oven at 105 °C for 48 h for determination of DM and used to quantify DMI. Secondary subsamples were dried at 50 °C in a forced-air drying oven for 72 h and ground to pass through a 2-mm screen (Wiley Mill, Swedesboro, NJ) in preparation for nutrient analyses. An ANKOM 200 fiber analyzer (ANKOM Technology, Macedon, NY) was used to determine neutral detergent fiber (**NDF**) with the addition of sodium sulfide and *a*-amylase (Van Soest et al., 1991) and acid detergent fiber (**ADF**; Method: 973.18; AOAC, 1995). Diet samples, pellet samples, NDF residue, and ADF residue were analyzed for crude protein (CP) via combustion (VarioMax Cube; Elementar Americas Inc., Ronkonkoma, NY; Method: 972.43, AOAC, 1995) to provide diet and pellet content of CP, neutral detergent insoluble CP (NDICP), and acid detergent insoluble CP (ADICP). Crude fat was determined using an ANKOM XT15 analyzer (ANKOM Technology; Komarek et al., 2004) with petroleum ether. Lignin (Method: 973.18; AOAC, 1995) was evaluated on ADF residue using 72% H_2_SO_4_ at ambient temperature and constant rotation for 3 h in the ANKOM Daisy^II^ system (ANKOM Technology). Ash (% of DM; Method: 942.05, AOAC, 1995) was quantified on feed and pellet samples using a gravity convection oven at 600 °C for 8 h. Nutrient composition was used to calculate total digestible nutrients (**TDN**; Weiss et al., 1992) which was then used to calculate digestible energy (Crampton et al., 1957; NASEM, 2016). Digestible energy was used to estimate ME and ultimately NE_m_ and NE_g_ using equations from Galyean et al. (2016).

### Energy calculations

Refer to Table 3 for an overview of equations utilized to calculate gaNE_m_ and gaNE_g_. HP was calculated in four ways utilizing gas flux data from the AHCS. First, RQ and CO_2_ emissions were used to calculate HP using the equation of Kaufmann et al. (2011; Kaufmann-HP). Additionally, HP was calculated using the Brouwer (1965) equation with one of three adjustments: the adjustment with N excretion omitted (No-UN HP), utilizing daily N intake and equations from Waldrip et al. (2013) to estimate UN (Waldrip-UN HP), or utilizing SUN and equations from Kohn et al. (2005) to estimate UN (Kohn-UN HP). ME (Mcal/d) was calculated using the mathematical and thermodynamic relationship between HP (Brouwer, 1965) and RE (NRC, 1984) for all four methods used to calculate HP. Constituents of the RE equation include empty BW (**EBW**) and empty body gain (**EBG**), which were calculated by shrunk BW (**SBW**; 96.0% live BW) and converting to EBW assuming EBW is 89.1% of SBW (NASEM, 2016). Shrunk ADG was calculated over the 80-d feeding period and was adjusted to EBG assuming EBG is 95.6% of shrunk ADG (NRC 1984; Oltjen and Garrett, 1988). Next, DMI was used to quantify ME per kilogram of DMI (Mcal ME/kg DM) and then dietary gaNE_m_ and gaNE_g_ were estimated using Galyean et al. (2016) cubic equations.

**Table 3.** Equations utilized to evaluate gaNE_m_ and gaNE_g_ using gas flux data from an automated head chamber system and performance data from feedlot steers consuming one of three finishing rations

Variable^1^^,^^2^	Equation	Source
HP, Mcal/d	$\;(3.866\times O_{2}+1.200\times \;CO_{2}\;-0.518\times CH_{4}-1.431\times \;UN)/1,000$	Brouwer (1965)
HP, Mcal/d	$4.96+(16.07/RQ)\times CO_{2}$	Kaufmann et al. (2011)
RE, Mcal/d	$0.0635\times EBW^{0.75}\;\times \;EBG^{1.097}$	NRC (1984)
ME, Mcal/kg DMI	(HP+RE)/DMI	NRC (1984)
NE_m_, Mcal/kg DM	$1.104\times ME-0.0946\times ME^{2}+0.0065\times ME^{3}-0.7783$	Galyean et al. (2016)
NE_g_, Mcal/kg DM	$1.1376\times ME-0.1198\times ME^{2}+0.0076\times ME^{3}-1.2979$	Galyean et al. (2016)

^1^HP = heat production; RE = recovered energy; NE_m_ = net energy for maintenance; NE_g_ = net energy for gain; O_2_ = oxygen (L/d); CO_2_ = carbon dioxide (L/d) CH_4_ = methane (L/d); UN = urinary nitrogen (g/d); EBW = empty body weight; EBG = empty body gain; DMI = dry matter intake; ME = metabolizable energy (Mcal/kg DM); DM = dry matter.

^2^EBW calculated as 89.1% of shrunk body weight (96.0% live weight) and EBG calculated as 95.6% of shrunk ADG over an 80-d feeding period.

Finally, dietary NE_m_ and NE_g_ were estimated in one of two ways by incorporating live and carcass performance data into the Zinn et al. (2008) quadratic performance-based equations (Table 4). As part of the method, required NE_m_ was calculated using average 80-d shrunk BW (SBW) whereas required NE_g_ was calculated using 80-d shrunk weight gain and equivalent SBW (EQSBW). Equivalent SBW was calculated by adjusting SBW to the BW equivalent of NRC (1984) medium-framed steers at 27.8% empty body fat (**EBF**; 428 kg) using either final shrunk BW (**FSBW**) from live animal performance data or adjusted-final BW (**AFWB**) from carcass data. Calculations for AFBW begin by estimating EBF (%) which is scaled to the FSBW at which point carcasses are expected to contain 28.0% EBF (Guiroy et al., 2001). It has been suggested that utilizing AFBW instead of FSBW would provide better estimates of paNE_m_ and paNE_g_ (Owens and Hicks, 2019). The paNE_m_ and paNE_g_ estimates using EQSBW and FSBW from live performance data are designated as live estimated paNE_m_ (live paNE_m_) and paNE_g_ (live paNE_g_). Dietary energy values derived from EQSBW implementing AFBW are denoted as carcass-estimated paNE_m_ (carcass paNE_m_) and paNE_g_ (carcass paNE_g_).

**Table 4.** Equations utilized to evaluate paNE_m_ and paNE_g_ using live performance or carcass data from feedlot steers consuming one of three finishing rations

Variable^1^^,^^2^^,^^3^^,^^4^^,^^5^	Equation	Source
NE_m_, Mcal/kg DM	$\;(b\pm \;\sqrt{b^{2}-4ac})/2a$	Zinn et al. (2008)
NE_g_, Mcal/kg DM	$0.877\;\times \;NE_{m\;\;\;}-0.41$ 0	Zinn et al. (2008)
*a*	0.877 × DMI	Zinn et al. (2008)
*b*	$-1\times [(0.877\times [-NE_{m\;\;\;}\;required])+(-0.410\times DMI)+(-NE_{g}\;required)]$	Zinn et al. (2008)
*c*	$\left(-\;0.410)\times (-NE_{m\;\;\;}\;required\right)$	Zinn et al. (2008)
NE_m_ required, Mcal/d	$Average\;\;\;SBW^{0.75}\times 0.077\;Mcal$	Zinn et al. (2008)
NE_g_ required, Mcal/d	$0.0557\times EQSBW^{0.75}\times SWG^{1.097}$	Zinn et al. (2008)
EQSBW, kg	Average SBW × (SRW/FSBW or AFBW)	NASEM (2016)

^1^NE_m_ = net energy for maintenance; NE_g_ = net energy for gain; DMI = dry matter intake; SBW = shrunk body weight; EQSBW = equivalent shrunk body weight; SWG = shrunk weight gain; SRW = standard reference weight; FSBW = final shrunk body weight; AFBW = adjusted-final body weight.

^2^SBW calculated as 96% of live weight and SWG calculated from initial and final SBW over an 80-d feeding period.

^3^SRW obtained from NASEM (2016) based on medium-framed steers from NRC (1984) reference database.

^4^AFBW is appraised final body weight at 28.0% empty fat as calculated from equations by Guiroy et al. (2001).

^5^EQSBW incorporates either final SBW or AFBW from live or carcass data, respectively.

### Statistical analyses

Analysis of variance (**ANOVA**) was conducted utilizing the Fit Model procedure of JMP Pro 16.0 (SAS Institute, Cary, NC) to determine the main effect of methodology on HP, UN, dietary NE_m_, and dietary NE_g_. Individual animal served as the experimental unit for all analyses. When applicable, least-square means were separated using Tukey–Kramer adjustments for multiple comparisons. Least-square mean differences were considered statistically significant if *P *< 0.05 and as having a tendency toward significance if 0.05 ≤* P *< 0.10.

Method agreement between NE_m_ and NE_g_ calculated by Kaufmann-HP, No-UN HP, Waldrip-UN HP, and Kohn-UN HP and live and carcass paNE_m_ and paNE_g_ were analyzed using R (v.4.1.0; R Core Team, 2021). Furthermore, method agreement between Kaufmann-HP estimated daily NE_m_ and NE_g_ intake values were compared with live paNE_m_ and paNE_g_ determined values. This assessment was made to compare a scenario where DMI is not known. Accordingly, only Kaufmann-HP method was assessed as in scenarios where DMI is unknown, daily UN excretion will be similarly unknown. Live paNEm and paNEg were only assessed for this analysis because carcass data will also likely be unknown in scenarios where DMI is unknown, such as in pasture. Firstly, daily ME intake was regressed on daily NE_m_ and NE_g_ values to determine an equation to convert daily ME intake to daily NE_m_ and NE_g_ intake. For all comparisons, method precision was assessed by Pearson’s correlation coefficient (*r*) using the “cor.test” function of base *R*. We suggest that high, moderate, and low precisions are characterized by *r* values of ≥0.90, ≥0.70 and <0.90, and <0.70, respectively. Accuracy and agreement between the methods were investigated using the “CCC” function of the “DescTools” package (Signorell et al., 2020). This function provides Lin’s concordance correlation coefficient (**CCC**), which is a function of a bias correction factor (Cb) and *r* (Lin., 1989, 2000). The Cb is calculated using two items. The first is termed scale shift, which is the ratio of the standard deviation between the two method estimates, and the second is termed location shift, which is analogous to the mean bias. In essence, the Cb is a measure of how far the two methods deviate from the line of unity, i.e., a one-to-one relationship with an intercept of 0. As such, Cb is a measure of accuracy. We propose that high, moderate, and low accuracy are characterized by Cb values of > 0.90, between 0.70 and < 0.90, and < 0.70, respectively. As CCC is calculated from a measure of accuracy (Cb) and precision (*r*), CCC can be considered a measure of agreement between the two methods. Lin’s CCC ranges from −1 to 1, similar to *r*; however, only values close to 1 indicate agreement. Also, like *r*, CCC requires the researcher or reader to define what values they consider to be adequate. For the purposes of this investigation, we suggest values of CCC for no agreement as <0, slight agreement as 0 to 0.39, moderate agreement as 0.40 to 0.59, adequate agreement as 0.60 to 0.80, and excellent agreement as > 0.80, which is similar to those suggested by Marshall et al. (2021). Next, root mean square error expressed as a percent of paNE_m_ or paNE_g_ was calculated using the “RMSE” function of the “DescTools” package (Signorell et al., 2020). Finally, the mean bias and slope bias between paNE_m_ and paNE_g_ and gaNE_m_ and gaNE_g_ for each of the four HP calculation methods explored was determined by regressing the mean-centered AHCS estimates with the residuals (i.e., performance-adjusted estimates minus AHCS estimates) as suggested by St-Pierre (2001).

## Results

Refer to Table 5 for an overview of observed performance, calculated RE, gas production, and values used to calculate UN. On average, cattle visited the AHCS units 1.6 times per day to provide an average of 128 visits per steer throughout the duration of the experiment. Urinary N was 24.3% greater (*P *< 0.01) when estimated by N intake calculated using the equation of Waldrip et al. (2013) compared with using SUN and the equation of Kohn et al. (2005; Table 6). Yet, there were no differences in HP (*P *= 0.99) when accounting for UN using No-UN HP, Waldrip-UN HP, or Kohn-UN HP (Table 6). Additionally, HP was not different when calculated using Kaufmann-HP compared to all other HP values and methods that accounted for UN (*P *= 0.99). There were no differences in estimated dietary NE_m_ when compared to carcass paNE_m_ and live paNE_m_ to gaNE_m_ generated using any HP value (*P *= 0.49). Similarly, there were no differences in estimates for dietary NE_g_ when compared to NE_g_ values generated using all other methods (*P *= 0.39).

**Table 5.** Observed cumulative performance, gas flux, SUN, urinary N excretion and N intake of steers fed one of three finishing rations and used to determine paNE_g_ and paNE_g_ or gaNE_m_ and gaNE_g_

Item^1^	Average	Minimum	Maximum	SD
Initial BW, kg	525	459	592	29.8
Final BW, kg	681	583	738	39.5
ADG, kg/d^2^	1.96	1.11	2.38	0.282
Pellet DMI, kg/d	0.62	0.20	0.88	0.131
TMR DMI, kg/d	10.09	7.58	11.77	0.915
Total DMI, kg/d	10.71	8.33	12.49	0.883
RE, Mcal/d^3^	13.15	6.76	16.74	2.210
CO_2_ emission, kg/d^4^	10.25	6.57	11.70	0.867
CH_4_ emission, kg/d^4^	0.151	0.111	0.208	0.2274
O_2_ consumption, kg/d^4^	6.84	4.76	7.84	0.577
SUN, mg/dL	8.31	5.40	12.97	1.49
UN excretion—Kohn, g/d	64.1	34.8	104.4	13.59
NI, g/d	79.67	54.13	97.26	10.558
UN excretion—Waldrip, g/d	79.7	54.1	97.3	10.56

^1^SD = standard deviation; BW = body weight; ADG = average daily gain; DMI = dry matter intake; RE = recovered energy; CO_2_ = carbon dioxide; CH_4_ = methane; O_2_ = oxygen; SUN = serum urea nitrogen; UN—Kohn = urinary nitrogen calculated from Kohn et al. (2005); NI = nitrogen intake; UN—Waldrip = urinary nitrogen calculated from Waldrip et al. (2013).

^2^ADG calculated using initial and final un-shrunk BW during an 80-d feeding trial.

^3^RE calculated from equations described by NRC (1984).

^4^Determined using an automated head chamber system (GreenFeed; C-Lock).

**Table 6.** Least-square means and statistical testing for the difference of methods to determine HP, UN, and paNE_m_ and paNE_g_ using live or carcass data

	Methodology							
Item	Kaufmann HP^1^	No-UN HP^2^	Kohn-UN HP^3^	Waldrip-UN HP^4^	Live pa^5^	Carcass pa^6^	Pooled SEM^7^	*P* value^8^
HP, Mcal/d	24.57	24.65	24.56	24.54	—	—	0.279	0.99
UN, g/d	—	—	64.10	79.67	—	—	1.666	<0.01
NE_m_, Mcal/kg DMI^9^	2.24	2.24	2.24	2.24	2.23	2.31	0.194	0.49
NE_g_, Mcal/kg DMI^9^	1.55	1.55	1.55	1.55	1.55	1.62	0.170	0.39

^1^HP estimated using RQ utilizing equation from Kaufmann et al. (2011).

^2^Urinay N omitted from Brouwer (1965) HP equation.

^3^SUN utilized to estimate UN from equation by Kohn et al. (2005) when estimating HP using Brouwer (1965) equation.

^4^Nitrogen intake utilized to estimate UN from equation by Waldrip et al. (2013) when estimating HP using Brouwer (1965) equation.

^5^Live pa incorporated, BW, ADG and DMI to estimate NE_m_ and NE_g_.

^6^Carcass pa method incorporated an appraisal of EBF from Guiroy et al. (2001) where final body weight was adjusted to 28.0% EBF (AFBW) in addition to utilizing live performance to estimate NE_m_ and NE_g_.

^7^Standard error of the mean.

^8^ANOVA *P* value.

^9^NE_m_ and NE_g_ estimated using HP, recovered energy from NRC (1984), and Galyean et al. (2016) or from Zinn et al. (2008) equations whereas the carcass pa method incorporated an appraisal of EBF from Guiroy et al. (2001) where final body weight was adjusted to 28.0% EBF (AFBW) in addition to utilizing live performance.

Analyses for precision, agreement, bias, and RMSE for estimates of dietary NE_m_ are outlined in Table 7 and dietary NE_g_ in Table 8. Dietary NE_m_ calculated from Kaufmann-HP, HP accounting for urine N (Waldrip-UN HP or Kohn-UN HP), or HP with no adjustment for urinary N (No-UN HP) all had high precision (*r* ≥ 0.90), high accuracy (Cb ≥ 0.92), and excellent agreement (CCC ≥ 0.83) when analyzed against carcass paNE_m_. When the gas-flux derived values were compared to live paNE_m_ there were also high precision (*r* = 0.91), high accuracy (Cb = 1.00), and excellent agreement (CCC = 0.91), regardless of adjustment for UN. However, average RMSE (as a % of respective paNE_m_) was 38.6% higher for estimates of dietary NE_m_ when carcass paNE_m_ was compared to live paNE_m_. As shown in Table 7, there was significant mean bias for Kaufmann-HP (0.07; *P *< 0.01), No-UN (0.07; *P *< 0.01), Waldrip-UN HP (0.08; *P *< 0.01), and Kohn-UN HP (0.07; *P *< 0.01) when each gaNE_m_ value was compared to carcass paNE_m_, yet there was no slope bias (*P *≥ 0.63) for any estimate of gaNE_m_. However, there was no evidence of mean (*P *≥ 0.46) or slope bias (*P *≥ 0.14) for all methods used to generate dietary gaNE_m_ values when compared to live paNE_m_.

**Table 7.** Comparative statistics of dietary NE_m_ estimated using one of four methods incorporating gas flux from an automated head chamber system or utilizing paNE_m_

	Kauffman HP^2^	Brouwer (1965) HP equation		
Item^1^		No-UN HP^3^	Kohn-UN HP^4^	Waldrip-UN HP^5^
Live pa^6^				
CCC	0.91	0.91	0.91	0.91
Cb	1.00	1.00	1.00	1.00
*r*	0.91	0.91	0.91	0.91
RMSE, %	3.43	3.45	3.43	3.43
Mean bias	−0.002	−0.008	−0.002	−0.001
* P* value	0.83	0.46	0.85	0.95
Slope bias	−0.09	−0.09	−0.09	−0.09
* P* value	0.15	0.14	0.14	0.14
Carcass pa^7^				
CCC	0.83	0.84	0.84	0.83
Cb	0.92	0.93	0.92	0.92
*r*	0.90	0.91	0.91	0.91
RMSE, %	4.79	4.63	4.80	4.83
Mean bias	0.07	0.07	0.07	0.08
* P* value	<0.01	<0.01	<0.01	<0.01
Slope bias	−0.03	−0.03	−0.03	−0.03
* P* value	0.65	0.64	0.63	0.63

^1^CCC = Lin’s concordance correlation coefficient (Lin., 1989); *r* = Pearson’s correlation coefficient; Cb = bias correction factor; RMSE = root mean square error; RMSE, % = RMSE as a percent of live pa or carcass pa.

^2^HP estimated using RQ utilizing equation from Kaufmann et al. (2011).

^3^Urinay N omitted from Brouwer (1965) HP equation.

^4^SUN utilized to estimate UN from equation by Kohn et al. (2005) when estimating HP using Brouwer (1965) equation.

^5^Nitrogen intake utilized to estimate UN from equation by Waldrip et al. (2013) when estimating HP using Brouwer (1965) equation.

^6^Live pa incorporated BW, ADG and DMI to estimate NE_m_ and NE_g_.

^7^Carcass pa method incorporated an appraisal of EBF from Guiroy et al. (2001) where final body weight was adjusted to 28.0% EBF (AFBW) in addition to utilizing live performance to estimate NE_m_ and NE_g_.

**Table 8.** Comparative statistics of estimated dietary NE_g_ using one of four methods incorporating gas flux from an automated head chamber system or utilizing paNE_g_

	Kauffman HP^2^	Brouwer (1965) HP equation		
Item^1^		No-UN HP^3^	Kohn-UN HP^4^	Waldrip-UN HP^5^
Live pa^6^				
CCC	0.91	0.91	0.91	0.91
Cb	1.00	1.00	1.00	1.00
*r*	0.91	0.91	0.91	0.91
RMSE, %	4.25	4.26	4.26	4.26
Mean bias	0.0015	−0.003	0.003	0.002
* P* value	0.88	0.73	0.76	0.85
Slope bias	−0.05	−0.05	−0.05	−0.05
* P* value	0.42	0.42	0.40	0.41
Carcass pa^7^				
CCC	0.82	0.83	0.82	0.82
Cb	0.91	0.92	0.91	0.91
*r*	0.91	0.91	0.91	0.91
RMSE, %	6.12	5.92	6.18	6.13
Mean bias	0.07	0.06	0.07	0.07
* P* value	<0.01	<0.01	<0.01	<0.01
Slope bias	0.01	0.01	0.008	0.009
* P* value	0.88	0.88	0.90	0.90

^1^CCC = Lin’s concordance correlation coefficient (Lin., 1989); *r* = Pearson’s correlation coefficient; Cb = bias correction factor; RMSE = root mean square error; RMSE, % = RMSE as a percent of live pa or carcass pa.

^2^HP estimated using RQ utilizing equation from Kaufmann et al. (2011).

^3^Urinay N omitted from Brouwer (1965) HP equation.

^4^SUN utilized to estimate UN from equation by Kohn et al. (2005) when estimating HP using Brouwer (1965) equation.

^5^Nitrogen intake utilized to estimate UN from equation by Waldrip et al. (2013) when estimating HP using Brouwer (1965) equation.

^6^Live pa incorporated BW, ADG and DMI to estimate NE_m_ and NE_g_.

^7^Carcass pa method incorporated an appraisal of EBF from Guiroy et al. (2001) where final body weight was adjusted to 28.0% EBF (AFBW) in addition to utilizing live performance to estimate NE_m_ and NE_g_.

As expected, trends for precision, accuracy, and agreement for estimates of NE_g_ were similar to NE_m_ since the same ME was utilized in Galyean et al. (2016) equations to estimate dietary NE_m_ and NE_g_. Statistical values for precision (*r* ≥ 0.91), accuracy (Cb ≥ 0.91), and agreement (CCC ≥ 0.82) were high for all gaNE_g_ values, regardless of HP, when compared to carcass paNE_g_ values. Once again, when dietary NE_g_ values were generated from any method to estimate HP for live paNE_g_, there was high precision (*r* = 0.91), accuracy (Cb = 1.00), and agreement (CCC = 0.91). However, RMSE (% of respective paNE_g_) values were, on average, 39.0% lower for all estimates of gaNE_g_ when compared to live paNE_g_ as opposed to carcass paNE_g_. Moreover, mean bias was evident for Kaufmann-HP (0.07; *P *< 0.01), No-UN HP (0.06; *P *< 0.01), Waldrip-UN HP (0.07; *P *< 0.01), and Kohn-UN HP (0.07; *P *< 0.01) when compared to carcass paNE_g_ yet there was no apparent slope bias (*P *≥ 0.88). In addition, there was no mean (*P ≥ *0.73) or slope bias (*P ≥ *0.40) when metrics to quantify HP and subsequent dietary NE_g_ were compared to live paNE_g_.

**Figure 1.**
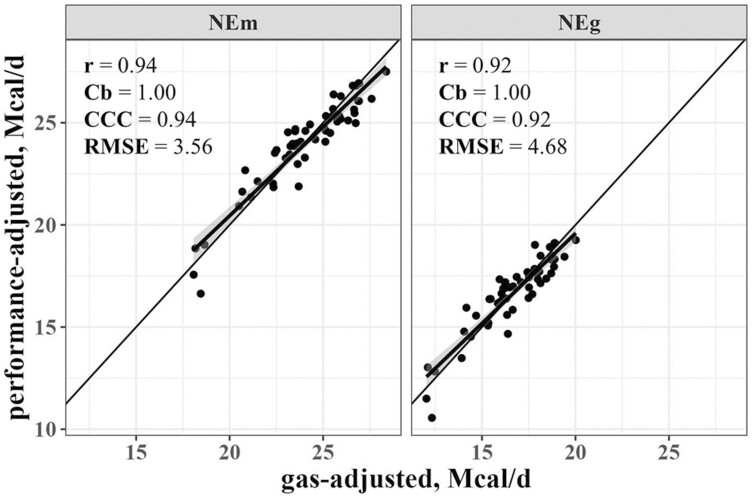
Agreement between daily NE_m_ and NE_g_ estimated based on performance-adjusted or gas-adjusted methodology. Pearson’s correlation (*r*) was used as a measure of precision, bias correction factor (Cb) as a measure of accuracy, Lin’s CCC as a measure of agreement, and root mean square error as a percent of the mean performance-adjusted values.


Finally, Figure 1 presents the scenario where DMI is unknown and comparison were made based on daily NE_m_ and NE_g_ intakes (Mcal/d) rather than NE_m_ and NE_g_ concentrations (Mcal/kg DM). We first needed to determine regression equations to convert ME intake to NE_m_ and NE_g_ intakes. These equations were

$$\begin{array}{l} NE_{m}(Mcal/d)=0.6643\;ME\;intake(Mcal/d)-1.0539\;(R^{2}=1.00) \end{array}$$ (1)

$$\begin{array}{l} NE_{g}(Mcal/d=0.5152\times ME\;\;\;intake(Mcal/d)-2.8061\;(R^{2}=0.97) \end{array}$$ (2)

The daily NE_m_ and NE_g_ intake values derived from the AHCS were then calculated using equations 1 and 2, respectively. There was high precision (*r* ≥ 0.92), accuracy (Cb = 1.00), and agreement (CCC ≥ 0.92) between daily NE_m_ and NE_g_ intakes estimated using daily ME intake (derived from the AHCS and observed performance) and equations 1 and 2, compared with those estimated by live paNE_m_ and paNE_g_. Accordingly, there were low RMSE values for NE_m_ (3.56%) and NE_g_ (4.68%).

## Discussion

The objective of this experiment was to compare estimates of dietary net energy values from performance data using live or carcass data with values estimated from gas flux data and calculated recovered energy, using four different means of calculating HP. Another objective of this experiment was to validate estimated energy values derived from gas flux data by evaluating precision, accuracy, and agreement against performance-adjusted dietary net energy values. The results presented herein suggest that gas flux data from an AHCS utilized to calculate HP and paired with estimates of RE can ultimately provide estimates of dietary NE_m_ and NE_g_ (gaNE_m_ and gaNE_g_) that agree with performance-adjusted values. The discussion below outlines results with support for these claims.

### Heat production

It is important to note that the estimation of HP provides the only source of variation in NE_m_ and NE_g_ values derived from gas flux measurements in the current experiment. Since RE is a function of observed performance, it is constant for each individual animal for all estimates of ME. Accordingly, HP is a key component in evaluating the efficacy of gas flux-based methodology. Calculating HP from the Brouwer (1965) equation has been utilized while ignoring the UN (g/d) component, as it is thought to account for less than 1% of total HP (Junghans et al., 2007; Kaufmann et al., 2011; Pereira et al., 2015). Although estimates of UN within the current study were 24.3% higher when using Waldrip-UN HP compared with Kohn-UN HP, there was no difference in HP between these methodologies. Furthermore, HP calculated by omitting the adjustment for UN (i.e., Kaufmann-HP and No-UN HP) were not different from the other options. This is because the adjustment for UN using the Brouwer (1965) equation only represented 0.37% and 0.45% of HP for the Kohn-UN HP and Waldrip-UN HP methodologies, respectively. In support, HP was curvilinear in sheep fed five levels of dietary CP where dietary inclusions between 10% and 15% dietary DM did not change HP (Cock et al., 1967). Dietary CP levels fed within the current study (10.77% to 12.51% CP, DM basis) may have been similar enough to prevent disparity in UN. Within the current study, Kohn-UN HP (0.37%) and Waldrip-UN HP (0.45%) accounted for less than 1% of total HP when evaluated against No-UN HP, which supports data presented by Cock et al. (1967). Collectively, data suggest that omitting the UN component of the Brouwer (1965) equation did not compromise the accuracy of computed HP in the current study. Further research may be warranted in cattle consuming a higher amount of CP than in the present study and in growing or grazing cattle to ensure ignoring UN is appropriate beyond the current research setting and feeding strategy.

Previous research (Junghans et al., 2007; Kaufmann et al., 2011) has suggested utilizing RQ and ignoring CH_4_ by mathematically rearranging the Brouwer (1965) HP equation. The proposed equation incorporates RQ and CO_2_ to estimate HP, yet the coefficients were adjusted by Kaufmann et al. (2011). Recently, this equation has been applied by researchers using the AHCS to measure gas flux (Pereira et al., 2015; Holder et al., 2022). We elected to implement the Kaufmann et al. (2011) equation that uses RQ as a secondary method to evaluate HP due to its frequent use in the literature, despite the lack of validation until the current experiment, to our knowledge. Interestingly, Holder et al. (2022) provided O_2_, CO_2_, and CH_4_ from AHCS which offered the opportunity to calculate HP using both Brouwer (1965) and Kaufmann et al. (2011) equations and their values were nearly identical. This supports the similarity between Kaufmann-HP and all other measures of HP observed in this experiment. Moreover, Hales et al. (2012) found a 26.4% increase in daily CH_4_ emission from cattle-fed diets with dry-rolled corn when compared to steam-flaked corn, yet this did not result in a difference in HP in sealed indirect calorimetry respiration chambers. Differences in CH_4_ production without influencing HP further support the notion that CH_4_ makes a relatively insignificant contribution to HP when calculated using the Brouwer (1965) equations. Similarly to UN, an adjustment for CH_4_ in the Brouwer (1965) equation accounted for less than 1% of total HP where correcting for CH_4_ accounted for 0.32% to 0.61% of No-UN HP and methods accounting for UN. This suggests that using RQ and the equation from Kaufmann et al. (2011) is an acceptable alternative to the Brouwer (1965) equation for calculating HP.

While using RQ is an acceptable method to calculate HP, it is necessary to quantify RQ from individual animals when using the Kaufmann et al. (2011) equation. Assuming a constant RQ of 1.0 in the Kaufmann et al. (2011) HP equation within the current analysis, HP was 3.8% higher than using individual RQ from gas flux data (i.e., Kaufmann-HP method). Moreover, a constant RQ of 1.05 resulted in a 6.7% increase in HP when compared with RQ from individual animals. Variation in RQ may be attributed to tissue energy balance, DMI, and energy metabolism (Armstrong and Blaxter, 1957). Often, a RQ of 1.0 or 1.05 is selected as a RQ of 1.0 is thought to represent the metabolic threshold of adipose accretion, but RQ may range from 0.7 to 1.2 depending on metabolic differences and dietary intake (Armstrong and Blaxter, 1957; Blaxter and Wainman, 1966). Yet, estimates of HP may be rendered inaccurate if O_2_ data is unavailable, as a reported correlation has been evaluated in grazing cattle where CO_2_ is only partially correlated (*r* = 0.72) to ME in the absence of O_2_ (Caetano et al., 2017). Thus, it is recommended that both CO_2_ and O_2_ are used in estimating individual animal RQ as opposed to assuming a constant RQ in cattle consuming a finishing diet.

### Method agreement

The data herein support the hypothesis that estimated dietary NE_m_ and NE_g_ are not different (*P *≥ 0.38) when comparing performance (i.e., live performance or carcass) to gas flux methodologies. Agreement (CCC ≥ 0.82) with either estimate for paNE_m_ and paNE_g_ supports the use of gas flux data from the AHCS to estimate HP to ultimately arrive at dietary NE_m_ and NE_g_ estimates. Carcass paNE_m_ was 3.6% higher while carcass paNE_g_ was 4.5% higher compared with live paNE_m_ and live paNE_g_, respectively. Additionally, RMSE was around 40% higher for carcass than live paNE_m_ and paNE_g_, respectively. These results may suggest that utilizing live performance data provides more accurate estimates of dietary NE_m_ and NE_g_. While this analysis is outside the bounds of this experiment, further investigation may be warranted since paNE_m_ and paNE_g_ is a tool commonly utilized by nutritionists to evaluate dietary energy (Owens and Hicks, 2019).

The greater agreement between gaNE_m_ and gaNE_g_ was higher when using live performance data. This discrepancy may be caused by using AFBW (Guiroy et al., 2001) as suggested by Owens and Hicks (2019) when calculating carcass paNE_m_ and paNE_g_. Replacing final SBW in the EQSBW equation with AFBW creates a scenario where metrics are scaled to the Garrett (1980) database using the standard reference weight of 428 kg for medium-framed steers from the NRC (1984) and then scaled again to 28.0% EBF when calculating AFBW. Since replacing final SBW with AFBW in the EQSBW scales the animal twice, it may result in the over-prediction of paNE_m_ and paNE_g_. Owens and Hicks (2019) suggested using AFBW in place of FSBW when calculating EQSBW. When AFBW was used in place of EQSBW, instead of as a component of the EQSBW equation, estimated paNE_m_ and paNE_g_ were biologically unrealistic (NE_m_ = 2.60 Mcal/kg and NE_g_ = 1.87 Mcal/kg). Moreover, when AFBW was replaced in each instance with FSBW, such as calculating average SBW or SWG, NE_m_ and NE_g_ values were unrealistically low based on observed performance (NE_m_ = 1.78 Mcal/kg and NE_g_ = 1.23 Mcal/kg). The average dietary energy values estimated from chemical analyses (NE_m_ = 2.22 Mcal/ kg and NE_g_ = 1.53 Mcal/kg) closely resemble paNE_m_ and paNE_g_ when using AFBW as replacement for final SBW within the EQSBW equation. These scenarios support our interpretation of applying AFBW to the EQSBW equation within the current dataset, but this may have been an erroneous assumption. However, utilizing EQSBW with final SBW generates paNE_m_ and paNE_g_ closely related to gaNE_m_ and gaNE_g_. When utilizing the Supplementary Data provided by Galyean et al. (2023) and Beck et al. (2023), the paNE_m_ and paNE_g_ values agreed more closely with wet-chemistry analyzed NE_m_ and NE_g_ values when AFBW replaced SBW in the EQSBW equation. This may indicate that the best methodology to calculate performance-adjusted energy values may be context-dependent, and we suggest related to days on feed. Further investigation is required to determine under what scenarios live and carcass paNE_m_ and paNE_g_ calculations are recommended. Regardless, it is concluded that researchers can deploy AHCS, calculate HP, estimate RE, and arrive at estimates of dietary NE_m_ and NE_g_ with excellent agreement to paNE_m_ and paNE_g_.

It is recognized that the application of gaNE_m_ and gaNE_g_ applies to research programs with access to AHCS, and more specifically, units with O_2_ sensors. Gas-flux methodology provides the opportunity to estimate dietary NE_m_ and NE_g_ while also evaluating potential differences or similarities in calculated HP, RE, and ME between treatments. Meta-analyses conducted by Owens and Hicks (2019) showed that paNE_g_ values were 6.2% to 7.8% lower than formulated NE_g_. Such variation may be a function of values used for individual ingredient NE_g_ when formulating rations, inaccurate measures of DMI or ADG, or differences in cattle NE_m_ requirements (Owens and Hicks, 2019). Calculating required NE_m_ is not a parameter in gas-flux methodology and between animal variation in NE_m_ requirements may be accounted for with gas measurements. Thus, it is postulated that gas flux may provide more accurate estimates in cattle with varying NE_m_ requirements, which may be a function of breed, sex, frame size, and previous or current plane of nutrition (Fox and Black, 1984; Fox et al., 1988). While this research provides the foundation for such claims, further investigation is required.

A potential setback for all methodologies is the duration of time required to gather live performance metrics suitable for calculating DMI and ADG. The minimum period of measurement for DMI and ADG has been established as 56 d on feed (BIF, 2010; Culbertson et al., 2015). Estimating dietary NE_m_ and NE_g_ throughout phases of the feedlot finishing period may provide more utility in research practice. Yet, as tissue accretion shifts from lean tissue to adipose growth (Simpfendorfer, 1973), estimates of dietary NE_m_ and NE_g_ from several phases may be rendered inaccurate due to changes in variation of NE_m_ requirements and a potential decrease in ADG (Fox et al., 1988). Moreover, utilizing carcass data to estimate dietary NE_m_ and NE_g_ is only viable for one dietary treatment for a set of cattle, serving as a potential limitation in a research setting. Performance metrics are components of gaNE_m_ and gaNE_g_ estimations whereas the equations of paNE_m_ and paNE_g_ are strictly dependent on such values. Therefore, it is worthwhile investigating if gaNE_m_ and gaNE_g_ can be estimated throughout several feeding phases to gain more utility in research projects, especially since dietary energy and HP can be evaluated. Regardless, a single estimate of paNE_m_ and paNE_g_ or gaNE_m_ and gaNE_g_ provides valuable insight into diets and cattle in feedlot research that can be incorporated into future experiments.

While the methodology incorporated within the current study supports agreement between performance and gas flux methodologies utilizing a subset of finishing cattle, further application may be useful in grazing cattle. Typically, in a research feedlot setting, collection of ADG and DMI data is relatively simple, allowing subsequent evaluation of paNE_m_ and paNE_g_ using quadratic solutions, which had strong agreement with gaNE_m_ and gaNE_g_ within this dataset. Yet, in grazing cattle, such as cows or stocker cattle, estimation of DMI is difficult, limiting the ability of estimating paNE_m_ and paNE_g_ using quadratic solutions. However, evaluating energy intake in grazing cattle can be achieved using HP and RE without DMI to estimate total ME intake (Mcal/d). Since estimates of RE only require average EBW and EBG, initial and final BW measurements may be satisfactory in estimating ME intake. Estimates of gaNE_m_ and gaNE_g_ from the current set of finishing cattle were obtained utilizing DMI data, which was available for incorporation into calculations. Yet, if the DMI is unknown, ME intake (Mcal/d) can be utilized to provide an estimate of energy intake or potentially be extrapolated to total gaNE_m_ or gaNE_g_ intake (Mcal/d) to potentially provide metrics of performance. This potential was explored using the current data and equations were developed to calculate daily NE_m_ and NE_g_ intake from daily ME intake values. When comparing NE_m_ and NE_g_ intake values using ME intake estimated from AHCS gas-flux and observed ADG in equations 1 and 2, respectively, with performance-adjusted NE_m_ and NE_g_ derived values, we determined excellent agreement (Figure 1). This analysis further highlights the ability to utilize AHCS to conduct energetic studies, even in scenarios where DMI is unknown. Furthermore, DMI could be extrapolated from ME intake and dietary ME content if it can be accurately quantified. It is important to note this requires accurate and reliable estimates of dietary ME content but could offer extended utility beyond a feedlot finishing scenario, as evaluation of estimated NE_m_ and NE_g_ in grazing cattle is often unreported given the difficulty of evaluating DMI. While this application currently remains a supposition, further research is warranted in grazing cattle to expand the potential utility of the gas flux methodology.

## Conclusions

Based on the results of this experiment, gas flux data from the AHCS was an acceptable method to estimate dietary NE_m_ and NE_g_ when compared to performance-adjusted methodologies and applied to a single dataset. Incorporating gas flux from AHCS affords researchers the opportunity to estimate HP in concert with dietary gaNE_m_ and gaNE_g_. Additionally, under our experimental conditions, it was unnecessary to adjust for UN and CH_4_ production when calculating HP. Since AHCS serves as a modified indirect respiration calorimetry system, gas data can be collected and utilized along with estimates of RE to estimate dietary NE_m_ and NE_g_, which are similar to live and carcass paNE_m_ and paNE_g_. While this may prove useful in a confined research setting, evaluating the efficacy of this methodology in grazing cattle may increase application in future research. Accordingly, further research is needed to investigate the use of the AHCS to conduct energetic studies in grazing systems. Researchers should determine whether paNE_m_ and paNE_g_ or gaNE_m_ and gaNE_g_ provide the most appropriate estimation of dietary energy within their experimental objectives and capabilities. It is worth noting the increased utility and data afforded by the AHCS as it can provide estimates of HP, gas emission or consumption, and estimates of dietary energy values.

## Supplementary Material

skae167_suppl_Supplementary_Materials

## Abbreviations

ADF: acid detergent fiber
ADG: average daily gain
ADICP: acid detergent insoluble crude protein
AFBW: adjusted-final body weight
AHCS: automated head chamber system
BW: body weight
Cb: bias correction factor
CCC: concordance correlation coefficient
CP: crude protein
DM: dry matter
DMI: dry matter intake
EBW: empty body weight
EQSBW: equivalent shrunk body weight
FSBW: final shrunk body weight
HP: heat production
ME: metabolizable energy
NDICP: neutral detergent insoluble crude protein
NE_g_: net energy for gain
NE_m_: net energy for maintenance
RE: recovered energy
RMSE: root mean square error
RQ: respiratory quotient
SRW: standard reference weight
SUN: serum urea nitrogen
TDN: total digestible nutrients
UN: urinary nitrogen

## References

[CIT0001] AOAC. 1995. Official methods of analysis. In: The analysis of agricultural materials. 16th ed. Arlington (VA): Association of Analytical Chemists.

[CIT0002] Armstrong, D., and K. Blaxter. 1957. The heat increment of steam-volatile fatty acids in fasting sheep. Br. J. Nutr. 11:247–272. doi:10.1079/BJN19570044 13460211

[CIT0003] Arthur, P., I. Barchia, C. Weber, T. Bird-Gardiner, K. Donoghue, R. Herd, and R. Hegarty. 2017. Optimizing test procedures for estimating daily methane and carbon dioxide emissions in cattle using short-term breath measures. J. Anim. Sci. 95:645–656. doi:10.2527/jas.2016.0700 28380597

[CIT0005] Beck, M. R., J. A. Proctor, Z. Kasuske, J. K. Smith, V. N. Gouvêa, C. L. Lockard, B. Min, and D. Brauer. 2023. Effects of replacing steam-flaked corn with increasing levels of malted barley in a finishing ration on feed intake, growth performance, and enteric methane emissions of beef steers. App. Anim. Sci. 39:525–534. doi:10.15232/aas.2023-02435

[CIT0006] Beck, M. R., L. R. Thompson, J. A. Proctor, R. R. Reuter, and S. A. Gunter. 2024. Recommendations on visit duration and number requirements for an automated head chamber system. J. Anim. Sci. 102:skae158. doi:10.1093/jas/skae158 38833215 PMC11190786

[CIT0004] Beef Improvement Federation (BIF). 2010. Guidelines for uniform beef improvement programs. 9th ed. Columbia (MO): Beef Improvement Federation. p. 24–27.

[CIT0009] Blaxter, K. L. 1962. The energy metabolism of ruminants. London (UK): Hutchinson Scientific and Technical.

[CIT0007] Blaxter, K., and F. Wainman. 1964. The utilization of the energy of different rations by sheep and cattle for maintenance and forfattening. J. Agric. Sci. 63:113–128. doi:10.1017/S002185960001515X

[CIT0008] Blaxter, K., and F. Wainman. 1966. The fasting metabolism of cattle. Br. J. Nutr. 20:103–111. doi:10.1079/BJN19660012 5939285

[CIT0010] Brouwer, E. 1965. Report of sub-committee on constants and factors. In: Proceedings of the 3rd Symposium on Energy Metabolism. Troon (Scotland): European Association of Animal Production Publication; p. 441–443.

[CIT0011] Caetano, M., M. Wilkes, W. Pitchford, S. Lee, and P. Hynd. 2017. Energy relations in cattle can be quantified using open-circuit gas-quantification systems. Anim. Prod. Sci. 58:1807–1813. doi:10.1071/AN16745

[CIT0012] Carstens, G., P. Mostyn, M. Lammoglia, R. Vann, R. Apter, and R. Randel. 1997. Genotypic effects on norepinephrine-induced changes in thermogenesis, metabolic hormones, and metabolites in newborn calves. J. Anim. Sci. 75:1746–1755. doi:10.2527/1997.7571746x 9222830

[CIT0013] Cock, L. M., B. R. Poulton, W. H. Hoover, and P. H. Knowlton. 1967. Dietary nitrogen effect on ruminant heat increment. J. Anim. Sci. 26:845–848. doi:10.2527/jas1967.264845x 6076878

[CIT0014] Crampton, E. W., L. E. Lloyd, and V. G. MacKay. 1957. The calorie value of TDN. J. Anim. Sci. 16:541–545. doi:10.1093/ansci/16.3.541

[CIT0015] Culbertson, M. M., S. E. Speidel, R. K. Peel, R. R. Cockrum, M. G. Thomas, and R. M. Enns. 2015. Optimum measurement period for evaluating feed intake traits in beef cattle. J. Anim. Sci. 93:2482–2487. doi:10.2527/jas.2014-8364 26020343

[CIT0016] Delfino, J. G., and G. W. Mathison. 1991. Effects of cold environment and intake level on the energetic efficiency of feedlot steers. J. Anim. Sci. 69:4577–4587. doi:10.2527/1991.69114577x 1752832

[CIT0017] Fox, D. G., and J. R. Black. 1984. A system for predicting body composition and performance of growing cattle. J. Anim. Sci. 58:725–739. doi:10.2527/jas1984.583725x

[CIT0018] Fox, D. G., C. J. Sniffen, and J. D. O’Connor. 1988. Adjusting nutrient requirements of beef cattle for animal and environmental variations. J. Anim. Sci. 66:1475–1495. doi:10.2527/jas1988.6661475x

[CIT0019] Galyean, M. L., N. A. Cole, L. O. Tedeschi, and M. E. Branine. 2016. Board invited review: Efficiency of converting digestible energy to metabolizable energy and reevaluation of the California net energy system maintenance requirements and equations for predicting dietary net energy values for beef cattle. J. Anim. Sci. 94:1329–1341. doi:10.2527/jas.2015-0223 27135993

[CIT0020] Galyean, M. L., K. E. Hales, and Z. K. Smith. 2023. Evaluating differences between formulated dietary net energy values and net energy values determined from growth performance in finishing beef steers. J. Anim. Sci. 101:1–8. doi:10.1093/jas/skad230 PMC10355367 37422728

[CIT0021] Garrett, W. 1980. Energy utilization by growing cattle as determined in 72 comparative slaughter experiments. In: Mount, L. E., editor. 8th Symposium on Energy Metabolism. Cambridge (UK): p. 3–7.

[CIT0022] Guiroy, P. J., D. G. Fox, L. O. Tedeschi, M. J. Baker, and M. D. Cravey. 2001. Predicting individual feed requirements of cattle fed in groups. J. Anim. Sci. 79:1983–1995. doi:10.2527/2001.7981983x 11518207

[CIT0023] Gunter, S. A., and M. R. Beck. 2018. Measuring the respiratory gas exchange by grazing cattle using an automated, open-circuit gas quantification system. Transl. Anim. Sci. 2:11–18. doi:10.1093/tas/txx009 32704685 PMC7200863

[CIT0024] Gunter, S. A., J. A. Bradford, and C. A. Moffet. 2017. Effects of mass airflow rate through an open-circuit gas quantification system when measuring carbon emissions. J. Anim. Sci. 95:475–484. doi:10.2527/jas.2016.0933 28177350

[CIT0025] Hales, K. E., N. A. Cole, and J. C. MacDonald. 2012. Effects of corn processing method and dietary inclusion of wet distillers grains with solubles on energy metabolism, carbon−nitrogen balance, and methane emissions of cattle. J. Anim. Sci. 90:3174–3185. doi:10.2527/jas.2011-4441 22585790

[CIT0026] Hammond, K. J., D. J. Humphries, L. A. Crompton, C. Green, and C. K. Reynolds. 2015. Methane emissions from cattle: Estimates from short-term measurements using a GreenFeed system compared with measurements obtained using respiration chambers or sulphur hexafluoride tracer. Anim. Feed Sci. Technol. 203:41–52. doi:10.1016/j.anifeedsci.2015.02.008

[CIT0027] Holder, A. L., M. A. Gross, A. N. Moehlenpah, C. L. Goad, M. Rolf, R. S. Walker, J. K. Rogers, and D. L. Lalman. 2022. Effects of diet on feed intake, weight change, and gas emissions in beef cows. J. Anim. Sci. 100:skac257. doi:10.1093/jas/skac257 35952719 PMC9527298

[CIT0028] Junghans, P., J. Voigt, W. Jentsch, C. C. Metges, and M. Derno. 2007. The 13C bicarbonate dilution technique to determine energy expenditure in young bulls validated by indirect calorimetry. Livest. Sci. 110:280–287. doi:10.1016/j.livsci.2006.11.009

[CIT0029] Kaufmann, L. D., A. Münger, M. Rérat, P. Junghans, S. Görs, C. C. Metges, and F. Dohme-Meier. 2011. Energy expenditure of grazing cows and cows fed grass indoors as determined by the 13C bicarbonate dilution technique using an automatic blood sampling system. J. Dairy Sci. 94:1989–2000. doi:10.3168/jds.2010-3658 21426990

[CIT0030] Kohn, R. A., M. M. Dinneen, and E. Russek-Cohen. 2005. Using blood urea nitrogen to predict nitrogen excretion and efficiency of nitrogen utilization in cattle, sheep, goats, horses, pigs, and rats. J. Anim. Sci. 83:879–889. doi:10.2527/2005.834879x 15753344

[CIT0031] Komarek, R., A. Komarek, and B. Layton. 2004. Evaluation of the rapid, high-temperature extraction of feeds, foods, and oilseeds by the ANKOM XT20 fat analyzer to determine crude fat content. Oil extraction and analysis: critical issues and comparative studies. Champaign (IL): AOCS Press; 39–68.

[CIT0032] Lin, L. I. -K. 1989. A concordance correlation coefficient to evaluate reproducibility. Biometrics 45:255–268. doi:10.2307/2532051 2720055

[CIT0033] Lin, L. I. -K. 2000. A note on the concordance correlation coefficient. Biometrics 56:324–325.

[CIT0034] Llonch, P., S. M. Troy, C. -A. Duthie, M. Somarriba, J. Rooke, M. J. Haskell, R. Roehe, and S. P. Turner. 2018. Changes in feed intake during isolation stress in respiration chambers may impact methane emissions assessment. Anim. Prod. Sci. 58:1011–1016. doi:10.1071/an15563

[CIT0035] Marshall, C. J., M. R. Beck, K. Garrett, N. Beale, and P. Gregorini. 2021. Evaluation of PEETER V1.0 urine sensors for measuring individual urination behavior of dairy cows. JDS Commun. 2:27–30. doi:10.3168/jdsc.2020-0019 36337287 PMC9623696

[CIT0036] NASEM. 2016. Nutrient requirements of beef cattle. 8th revised ed. Washington (DC): The National Academies Press.

[CIT0037] NRC. 1984. Nutrient requirements of beef cattle. Washington (DC): The National Academies Press.

[CIT0038] Oltjen, J., and W. Garrett. 1988. Effects of body weight, frame size and rate of gain on the composition of gain of beef steers. J. Anim. Sci. 66:1732–1738. doi:10.2527/jas1988.6671732x 3403405

[CIT0040] Owens, F. N., and R. B. Hicks. 2019. Can net energy values be determined from animal performance measurements? A review of factors affecting application of the California net energy system. Transl. Anim. Sci. 3:929–944. doi:10.1093/tas/txy130 32704857 PMC7252571

[CIT0039] Owens, F., W. Sharp, and D. Gill. 1984. Net energy calculation from feedlot performance data. Oklahoma Agricultural Experiment Station Miscellaneous Publication. Vol. 116. Stillwater (OK): 290–293.

[CIT0041] Pereira, A. B. D., S. A. Utsumi, C. D. Dorich, and A. F. Brito. 2015. Integrating spot short-term measurements of carbon emissions and backward dietary energy partition calculations to estimate intake in lactating dairy cows fed ad libitum or restricted. J. Dairy Sci. 98:8913–8925. doi:10.3168/jds.2015-9659 26506553

[CIT0042] Place, S. E., Y. Pan, Y. Zhao, and F. M. Mitloehner. 2011. Construction and operation of a ventilated hood system for measuring greenhouse gas and volatile organic compound emissions from cattle. Animals. 1:433–446. doi:10.3390/ani1040433 26486626 PMC4513474

[CIT0054] R Core Team. 2021. R: A language and environment for statistical computing. Vienna (Austria): R Foundation for Statistical Computing. http://www.r-project.org

[CIT0043] Signorell, K. A. A., A. Alfons, N. Anderegg, T. Aragon, A. Arppe, A. Baddeley, K. Barton, B. Bolker, H. W. Borchers, F. Caeiro, et al. 2020. DescTools: Tools for descriptive statistics. https://github.com/AndriSignorell/DescTools/

[CIT0044] Simpfendorfer, S. 1973. Relationship of body type and size, sex, and energy intake to the body composition of cattle. Cornell University. Dissertation

[CIT0045] St-Pierre, N. R. 2001. Invited Review: Integrating quantitative findings from multiple studies using mixed model methodology. J. Dairy Sci. 84:741–755. doi:10.3168/jds.S0022-0302(01)74530-4 11352149

[CIT0046] Van Soest, P. J., J. B. Robertson, and B. A. Lewis. 1991. Methods for dietary fiber, neutral detergent fiber, and nonstarch polysaccharides in relation to animal nutrition. J. Dairy Sci. 74:3583–3597. doi:10.3168/jds.S0022-0302(91)78551-2 1660498

[CIT0047] Vasconcelos, J. T., and M. L. Galyean. 2008. Technical note: Do dietary net energy values calculated from performance data offer increased sensitivity for detecting treatment differences? J. Anim. Sci. 86:2756–2760. doi:10.2527/jas.2008-1057 18539830

[CIT0048] Waldrip, H. M., R. W. Todd, and N. A. Cole. 2013. Prediction of nitrogen excretion by beef cattle: a meta-analysis. J. Anim. Sci. 91:4290–4302. doi:10.2527/jas.2012-5818 23825341

[CIT0049] Wedegaertner, T. C., and D. E. Johnson. 1983. Monensin effects on digestibility, methanogenesis and heat increment of a cracked corn-silage diet fed to steers. J. Anim. Sci. 57:168–177. doi:10.2527/jas1983.571168x 6885657

[CIT0050] Weiss, W. P., H. R. Conrad, N. R., St. Pierre. 1992. A theoretically-based model for predicting total digestible nutrient values of forages and concentrates. Anim. Feed Sci. Technol. 39:95–110. doi:10.1016/0377-8401(92)90034-4

[CIT0053] Zinn, R. A., and Y. Shen. 1998. An evaluation of ruminally degradable intake protein and metabolizable amino acid requirements of feedlot calves. J. Anim. Sci. 76:1280–1289. doi:10.2527/1998.7651280x 9621934

[CIT0052] Zinn, R. A., R. Barrajas, M. Montano, and R. A. Ware. 2003. Influence of dietary urea level on digestive function and growth performance of cattle fed steam-flaked barley-based finishing diets. J. Anim. Sci. 81:2383–2389. doi:10.2527/2003.81102383x 14552362

[CIT0051] Zinn, R., A. Barreras, F. Owens, and A. Plascencia. 2008. Performance by feedlot steers and heifers: daily gain, mature body weight, dry matter intake, and dietary energetics. J. Anim. Sci. 86:2680–2689. doi:10.2527/jas.2007-0561 18539825

